# Daily Collection of Self-Reporting Sleep Disturbance Data via a Smartphone App in Breast Cancer Patients Receiving Chemotherapy: A Feasibility Study

**DOI:** 10.2196/jmir.3421

**Published:** 2014-05-23

**Authors:** Yul Ha Min, Jong Won Lee, Yong-Wook Shin, Min-Woo Jo, Guiyun Sohn, Jae-Ho Lee, Guna Lee, Kyung Hae Jung, Joohon Sung, Beom Seok Ko, Jong-Han Yu, Hee Jeong Kim, Byung Ho Son, Sei Hyun Ahn

**Affiliations:** ^1^Seoul National University College of NursingSeoulRepublic of Korea; ^2^Department of SurgeryUniversity of Ulsan College of MedicineAsan Medical CenterSeoulRepublic of Korea; ^3^Department of EpidemiologySchool of Public Health and Institute of Health and EnvironmentSeoul National UniversitySeoulRepublic of Korea; ^4^Department of PsychiatryUniversity of Ulsan College of MedicineAsan Medical CenterSeoulRepublic of Korea; ^5^Department of Preventive MedicineUniversity of Ulsan College of MedicineAsan Medical CenterSeoulRepublic of Korea; ^6^Department of Emergency MedicineUniversity of Ulsan College of MedicineAsan Medical CenterSeoulRepublic of Korea; ^7^Medical Information Administration TeamAsan Medical CenterSeoulRepublic of Korea; ^8^Department of OncologyUniversity of Ulsan College of MedicineAsan Medical CenterSeoulRepublic of Korea

**Keywords:** mobile applications, self report, compliance, breast cancer

## Abstract

**Background:**

Improvements in mobile telecommunication technologies have enabled clinicians to collect patient-reported outcome (PRO) data more frequently, but there is as yet limited evidence regarding the frequency with which PRO data can be collected via smartphone applications (apps) in breast cancer patients receiving chemotherapy.

**Objective:**

The primary objective of this study was to determine the feasibility of an app for sleep disturbance-related data collection from breast cancer patients receiving chemotherapy. A secondary objective was to identify the variables associated with better compliance in order to identify the optimal subgroups to include in future studies of smartphone-based interventions.

**Methods:**

Between March 2013 and July 2013, patients who planned to receive neoadjuvant chemotherapy for breast cancer at Asan Medical Center who had access to a smartphone app were enrolled just before the start of their chemotherapy and asked to self-report their sleep patterns, anxiety severity, and mood status via a smartphone app on a daily basis during the 90-day study period. Push notifications were sent to participants daily at 9 am and 7 pm. Data regarding the patients’ demographics, interval from enrollment to first self-report, baseline Beck’s Depression Inventory (BDI) score, and health-related quality of life score (as assessed using the EuroQol Five Dimensional [EQ5D-3L] questionnaire) were collected to ascertain the factors associated with compliance with the self-reporting process.

**Results:**

A total of 30 participants (mean age 45 years, SD 6; range 35-65 years) were analyzed in this study. In total, 2700 daily push notifications were sent to these 30 participants over the 90-day study period via their smartphones, resulting in the collection of 1215 self-reporting sleep-disturbance data items (overall compliance rate=45.0%, 1215/2700). The median value of individual patient-level reporting rates was 41.1% (range 6.7-95.6%). The longitudinal day-level compliance curve fell to 50.0% at day 34 and reached a nadir of 13.3% at day 90. The cumulative longitudinal compliance curve exhibited a steady decrease by about 50% at day 70 and continued to fall to 45% on day 90. Women without any form of employment exhibited the higher compliance rate. There was no association between any of the other patient characteristics (ie, demographics, and BDI and EQ5D-3L scores) and compliance. The mean individual patient-level reporting rate was higher for the subgroup with a 1-day lag time, defined as starting to self-report on the day immediately after enrollment, than for those with a lag of 2 or more days (51.6%, SD 24.0 and 29.6%, SD 25.3, respectively; *P*=.03).

**Conclusions:**

The 90-day longitudinal collection of daily self-reporting sleep-disturbance data via a smartphone app was found to be feasible. Further research should focus on how to sustain compliance with this self-reporting for a longer time and select subpopulations with higher rates of compliance for mobile health care.

## Introduction

Electronic health (eHealth) can be defined as the practice of medicine and public health using information and communication technology (ICT), such as computers, mobile phones, and satellite communications [1]. The term mobile health (mHealth) has emerged as a subcategory of eHealth and is now used when the practice involves using wireless communications and especially mobile phones or smartphones, which are now used by more than 70% of the population in some countries, including the United Arab Emirates, South Korea, Saudi Arabia, and Singapore (as of 2013; see Multimedia Appendix 1). Although evidence regarding the value of incorporating mobile phone-based patient-reported outcome (PRO) data into cancer patients’ care is still in the embryonic stage, noteworthy to date are demonstrations of the feasibility of mHealth—and specifically the use of mobile phones—to assist in the collection of PROs on disease-related vital signs [2,3], treatment-related side effects [4], and possibly comprehensive psychological status [5].

Prior to the eHealth era, the collection of PROs in the oncology field was based simply on patient recall at clinic visits or by asking patients to keep paper diaries outside the clinic. However, patient recall is inherently inaccurate and plagued by potential bias and the actual compliance with keeping paper diaries according to protocols has proved to be much lower than was expected [6], thus undermining the rationale for using it. The advent of ICT, which can build survey systems with a broad range of clinical uses, has made it possible to capture PRO data in real time and broken through a key barrier to the use PRO data in the clinical-care setting. These kinds of electronic PROs (ePROs) from waiting rooms in the hospital [7,8], as well as from home [9], have reportedly been successfully collected with high mean compliance rates and patient satisfaction and are now increasingly used in routine outpatient cancer care to guide clinical decisions and enhance communication. However, most of the available evidence has been obtained from studies using tablets or desktop computers [7-9] and none of the evidence collected using mobile phones [2,4,10-12] involved the currently available smartphones that have unique functions such as push notification and flexible applications (apps). To the best of our knowledge, there is limited information regarding the feasibility and acceptability of smartphone-based collection of PROs for cancer patients receiving chemotherapy and in particular for daily data collections over long periods of time.

The primary objective of this study was to determine the feasibility of using a smartphone app to collect sleep disturbance-related data from breast cancer patients receiving chemotherapy, with overall and individual reporting rates. We also sought to determine whether the patients stopped responding via the app after a short time by calculating a longitudinal reporting rate per day over a period of 90 days. A secondary objective was to elucidate the variables associated with a higher compliance rate in order to identify the optimal subgroups to include in future studies of smartphone-based interventions.

## Methods

### Participant Recruitment

Patients who were planning to receive neoadjuvant chemotherapy for breast cancer were recruited for participation in this feasibility study when they were admitted to the Breast Cancer Center, Asan Medical Center, Seoul, South Korea, for 2 nights to evaluate the disease status for preoperative chemotherapy between March 2013 and July 2013. To be eligible for this study, patients had to indicate app avidity, be current iOS or Android smartphone users, and be able to read and understand Korean. In some cases, the app did not run properly due to unexpected incompatibility between the smartphones’ display specifications and the app.

**Figure 1:**
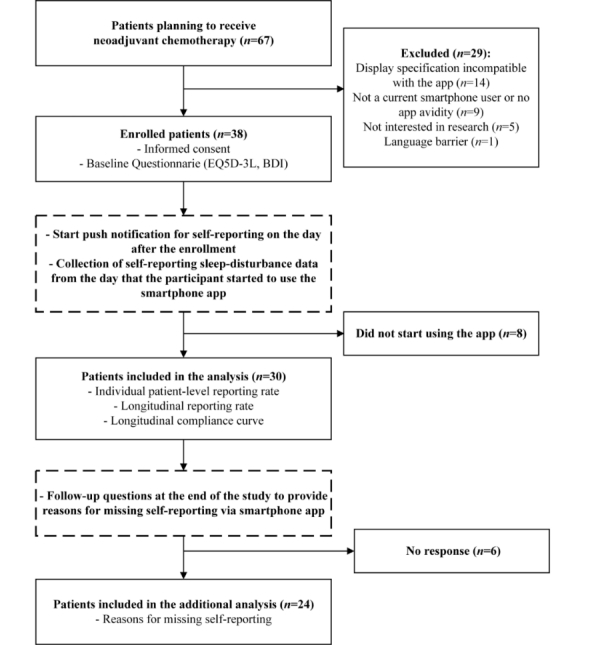
Study design and participant flow.

Among the 67 patients initially recruited for participation, 29 were excluded for the following reasons: incompatibility of the app with their smartphone’s display specifications (n=14), not a current smartphone user or no app avidity (n=9), not interested in the research (n=5), and language barrier (n=1). A total of 38 patients were enrolled and provided informed consent to participate (IRB no. 2012-0709). Of these, 8 patients were not included in the analysis performed in this feasibility study because they did not start to use the app or did not report sleep-disturbance symptoms at all after providing consent to participate at admission (Figure 1).

The participants were presented with information regarding the study and the app, called “Pit-a-Pat”, which was developed for both the iOS and Android platforms, and has been described previously [13]. A 30-minute interview with each patient at admission included the following items: downloading of the app; entering baseline demographics such as age, educational attainment, marital status, cohabitation status, and occupation into the app; instructions on how to log in to the app and report information about sleep disturbance (possibly caused by the breast cancer itself and the chemotherapy); and baseline survey of health-related quality of life (HRQOL) status as assessed using the EQ5D-3L questionnaire and depression status as assessed using Beck’s Depression Inventory (BDI).

After a 2-night admission for workup, the participants were discharged and scheduled to visit the outpatient clinic 7 days later (on average) to receive neoadjuvant chemotherapy. The workup results for neoadjuvant chemotherapy, such as regional lymph node metastasis, hormone receptor expression, and planned chemotherapeutic agents regimens, were also collected.

### Daily Collection of Symptoms via the App

**Figure 2:**
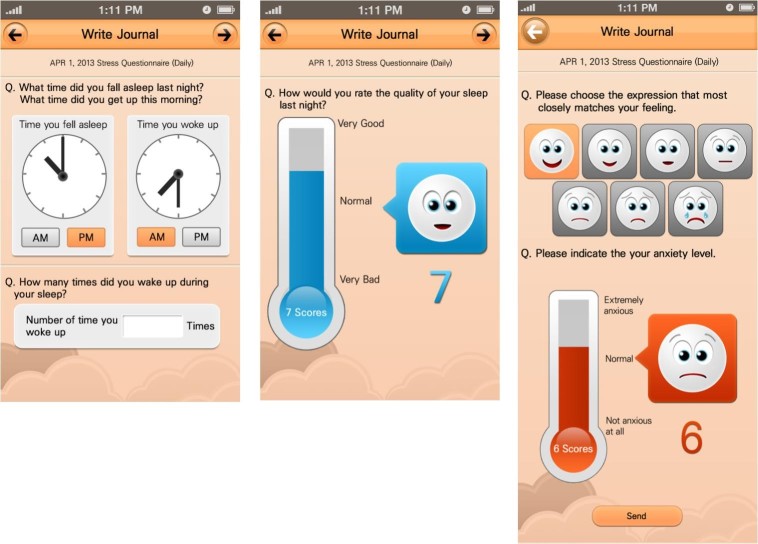
Screenshot of the app for self-reporting of sleep disturbance data.

Briefly, the app in this study was developed for cancer patients to self-report three kinds of health experience that may be caused by the effect of the diagnosis with breast cancer itself and the subsequent treatments: (1) sleep-disturbance symptoms related to mild depression, (2) acute symptoms related to cytotoxic chemotherapeutic agents, and (3) medication diary for antihormonal treatment such as tamoxifen and aromatase inhibitor. The sleep-disturbance symptoms related to mild depression were assessed using a six-item questionnaire: (1) time of falling asleep, (2) time of waking up, (3) number of awakenings during the total sleep period, (4) quality of total sleep on a 10-point rating scale, (5) present mood status on a 7-point rating scale, and (6) severity of present anxiety on a 10-point rating scale. The sleeping and waking times (in minutes) were stored as continuous variables. The 10-point rating scale for quality of total sleep and severity of present anxiety was displayed as a distress thermometer and the 7-point rating scale for mood status was displayed as faces with a range of expressions. The six items were displayed in three sequential pages as shown in Figure 2.

Participants were informed that they could start reporting their symptoms via the app at any time from the day following their provision of consent. Once the app was activated, push notifications were sent to the participants daily at 9 am and 6 pm, asking them to daily self-report sleep patterns, anxiety severity, and mood status via the smartphone app over a 90-day period. After entering appropriate answers to all six items without missing values, a participant could touch the send button and be considered as having completed a self-report of her sleep-disturbance symptoms on that specific day. The same push notification process was repeated for all participants during the study period, irrespective of their reporting rates. A database system collected the information about daily requests and inputs (ie, anonymized user ID, item ID, date and time of input, and input value), as well as baseline demographics.

The database system was connected to our electronic medical record system to enable clinicians to review the data. During the 90-day study period, clinicians could review the participants’ self-reporting data at the outpatient clinic at 3-week intervals, and managed them in the same way as the nonparticipating patients for the reported symptoms, since this was a study only related to feasibility. Participants were not provided with a smartphone for this study, instead being required to use their own device. Furthermore, they did not receive any reimbursement of expenses for extra data usage caused by utilization of the app, or any financial incentive for participating in the study.

At the end of the study period, the participants received a push notification requesting reasons for missing self-reporting, by choosing from the following possible responses: “The app didn’t work properly”, “I forgot”, “I didn’t think it was useful”, “I was too sick”, “I didn’t feel like it”, “It was inconvenient”, or “I was too busy”. Among the 30 participants who received 90 requests for self-reporting, 24 responded and their data were analyzed.

### Statistical Analysis

The feasibility of this self-reporting system was analyzed by calculating individual patient-level reporting rates, defined as the total number of days in which self-reporting was completed divided by 90 for each participant, and a longitudinal day-level reporting rate, defined as the total number of participants who completed self-reporting divided by 30 on a single specific day from the start of self-reporting. A cumulative longitudinal day-level reporting rate was also defined as the mean of all longitudinal day-level reporting rates from the starting day until the specified day. For example, the longitudinal day-level reporting rate at day 34 indicates the number of participants self-reporting on the 34^th^ day since the starting day divided by 30 and the cumulative longitudinal day-level reporting rate at day 34 indicates the mean value of all longitudinal day-level reporting rates from day 1 to day 34. A longitudinal compliance curve was plotted by connecting the 90 longitudinal day-level reporting rates for each day over the 90-day study period and a cumulative longitudinal compliance curve with 90 cumulative longitudinal day-level reporting rates over time. For the cumulative dropout probability curve, a dropout event was defined as when a cumulative longitudinal day-level reporting rate of a participant decreases first to below 28.6%, which means that she reported less than twice per week, which is almost the same as the weekly biodata collection.

For a priori threshold criteria for good compliance, prior feasibility studies in related contexts were reviewed [3,4,7-9,14] to determine whether this daily data-collection approach via smartphone app merited further development. On a reasonable threshold for feasibility, experts came to the conclusion that the previous evidence was insufficient because of small samples [3,4], use of a tablet, desktop computer, or conventional mobile phones rather than a current smartphone [3,4,7-9,14], and inclusion of patients with noncancerous chronic diseases [3]. Instead, we decided to explore the feasibility ourselves, focusing on the following characteristics of this study cohort: women with breast cancer, receiving chemotherapy, daily collection, smartphone with an app, and South Korea’s high degree of smartphone penetration. In addition, we planned to establish an appropriate cutoff for the higher compliance rate after describing the distribution of various reporting rates.

The chi-square test and *t* test were used to identify differences in variables between groups with higher and lower rates of compliance. Baseline patient variables of interest included age, educational attainment, occupation status, HRQOL status, BDI score, and interval from enrollment to first self-reporting. This study was performed in a cohort with uniform characteristics in terms of race, cancer type and stage, and Eastern Cooperative Oncology Group performance status. Except where stated otherwise, the data are presented as mean (SD) values and the threshold of statistical significance was set at *P*<.05. All statistical analyses were performed using SPSS version 12.0 (SPSS, Chicago, IL, USA).

## Results

### Participant Characteristics

Between March 2013 and July 2013, 67 patients were initially eligible for participation. However, 14 patients (20%, 14/67) could not participate in this study because of inherent technological limitations in the development of the app causing incompatibility with their smartphone’s display specifications. Among the 53 women who did not experience technical problems in using the app, 9 (17%, 9/53) showed no interest in either their smartphone or the app and 5 (9%, 5/53) refused to participate in this study because they were not interested in the research. Of the 38 eligible patients who were actually enrolled into this feasibility study, 8 (21%, 8/38) did not start to use the app. The final analysis thus included 30 patients (mean age 45 years, SD 6; range 36-65 years), all 30 of whom were Korean women who had been diagnosed with breast cancer within 4 weeks prior to enrollment; 73% (22/30) were 50 years old, 47% (14/30) had an educational attainment of college level or higher, and 43% (13/30) were currently employed. The baseline BDI and EQ5D-3L scores were 11.5 (SD 8.8) (range 0-35) and 0.92 (SD 0.09) (range 0.56-1.00), respectively. For tumor characteristics, lymph-node metastases were confirmed in 77% (23/30), 60% (18/30) were hormone-responsive breast cancer, and those with any kind of distant metastases were excluded. During the 90-day study period, all participants received adriamycin-based (87%, 26/30) or epirubicin-based (13%, 4/30) combinational chemotherapeutic agents, of whom 77% (23/30) received additional docetaxel because of positive lymph-node metastasis after this feasibility study (Table 1).

**Table 1:** Baseline demographics of patients (n=30).

Characteristic			Total (n=30)	Lower compliance rate (n=15)	Higher compliance rate (n=15)	*P*
			n (%)			
**Age**						
	Mean (SD) years		45 (6)	46 (5)	45 (8)	NS^a^
	Range, years		36-65	38-55	36-65	
	≤49 years		22 (73)	11 (73)	11 (73)	NS
	≥50 years		8 (27)	4 (27)	4 (27)	
**Level of educational attainment**						NS
	Up to high school		16 (53)	10 (67)	6 (40)	
	College or greater		14 (47)	5 (33)	9 (60)	
**Marital status**						NS
	Married		26 (87)	13 (87)	13 (86)	
	Single		1 (3)	0 (0)	1 (7)	
	Divorced		3 (10)	2 (13)	1 (7)	
**Cohabiting**						NS
	Yes		28 (93)	15 (100)	13 (87)	
	No		2 (7)	0 (0)	2 (13)	
**Occupation**						.03
	Yes (of any kind)		13 (43)	10 (67)	3 (20)	
	No (eg, stay-at-home mother)		17 (57)	5 (33)	12 (80)	
**BDI** ^b^ **score**						
	Mean (SD)		11.5 (8.8)	11.9 (8.5)	11.1 (9.4)	NS
	Range		0-35	1-33	0-35	
	≤15		21 (70)	10 (67)	11 (73)	NS
	≥16		9 (30)	5 (33)	4 (27)	
**HRQOL** ^c^ **with EQ5D-3L** ^d^						
	**EQ5D-3L VAS** ^e^ **score**					NS
		Mean (SD)	69.6 (16.1)	68.3 (16.0)	70.9 (16.6)	
		Range	38-99	45-95	38-99	
	**EQ5D-3L utility score**					NS
		Mean (SD)	0.92 (0.09)	0.92 (0.07)	0.91 (0.11)	
		Range	0.56-1.00	0.74-1.00	0.56-1.00	
	**Mobility**					NS
		No problems	29 (97)	15 (100)	14 (93)	
		Problems	1 (3)	0 (0)	1 (7)	
	**Self-care**					NS
		No problems	30 (100)	15 (100)	15 (100)	
		Problems	0 (0)	0 (0)	0 (0)	
	**Usual activities**					NS
		No problems	29 (97)	15 (100)	14 (93)	
		Problems	1 (3)	0 (0)	1 (7)	
	**Pain/discomfort**					NS
		No problems	19 (63)	11 (73)	8 (53)	
		Problems	11 (37)	4 (27)	7 (47)	
	**Anxiety/depression**					NS
		No problems	14 (47)	6 (40)	8 (53)	
		Problems	16 (53)	9 (60)	7 (47)	
**Lymph node metastasis**						NS
	Negative		7 (23)	4 (27)	3 (20)	
	Positive		23 (77)	11 (73)	12 (80)	
**Hormone receptor status**						NS
	Positive		18 (60)	6 (40)	12 (80)	
	Negative		12 (40)	9 (60)	3 (20)	
**Neoadjuvant chemotherapy regimen**						NS
	AC^f^#4		7 (23)	4 (27)	3 (20)	
	AC#4 followed by docetaxel#4		19 (64)	7 (46)	12 (80)	
	FEC^g^#3 followed by docetaxel#3		4 (13)	4 (27)	0 (0)	

^a^Not significant.

^b^BDI: Beck’s Depression Inventory.

^c^HRQOL: health-related quality of life.

^d^EQ5D-3L: EuroQol Five Dimensional Questionnaire.

^e^Visual analog scale.

^f^Combination of doxorubicin+cyclophosphamide chemotherapy.

^g^Combination of 5-fluorouracil+epirubicin+cyclophosphamide chemotherapy.

### Feasibility Analysis

**Figure 3:**
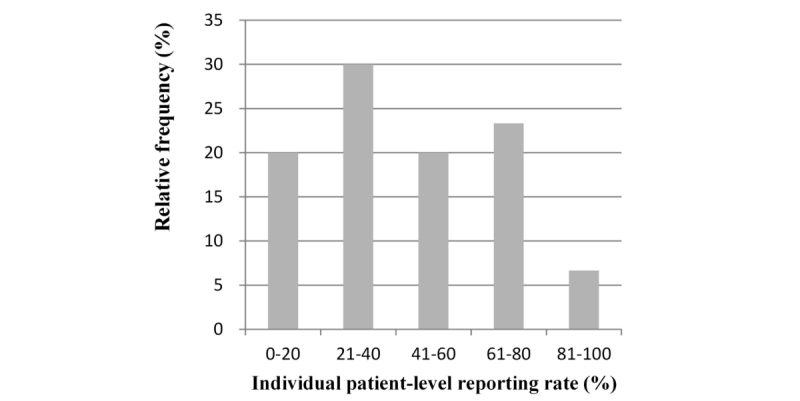
Distribution of individual patient-level reporting rates.

In total, 2700 daily push notifications were sent to the 30 participants via their smartphones during the 30-day study period; 1215 responses were received containing completed self-reporting sleep-disturbance data (overall compliance rate=45.0%; 1215/2700). The median individual patient-level reporting rates from the 30 participants was 41.1% (range 6.7-95.6%), with the following distribution: 6 (20%, 6/30), 9 (30%, 9/30), 6 (20%, 6/30), 7 (23%, 7/30), and 2 (7%, 2/30) ranging from 0% to 20%, 21% to 40%, 41% to 60%, 61% to 80%, and 81% to 100%, respectively (Figure 3).

**Figure 4:**
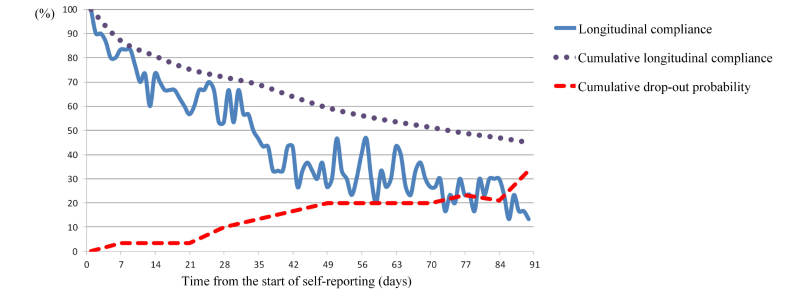
Changes in compliance over time.

The longitudinal day-level reporting rate on day 1 is theoretically 100%, because that day is the first day when all participants started to use the app. The longitudinal day-level reporting rates from day 1 to day 90 were calculated at daily intervals and plotted as a longitudinal compliance curve. The longitudinal compliance curve for this app decreased rapidly to about 50.0% at day 34 after the start of self-reporting, and continued to decrease steadily thereafter, reaching a nadir of 13.3% at day 90. The cumulative longitudinal compliance curve (representing overall compliance) revealed a steady decrease by about 50% at day 70, and continued to fall to 45% on day 90. Cumulative dropout probabilities showed that 10% (3/30) of all participants self-reported less than twice per week on average (cumulative longitudinal compliance rate <28.6%) during the first 28 days, 20% (6/30) during the first 56 days, and 33.3% (10/30) during the 90-day study period (Figure 4).

### Variables Associated With Compliance

The distribution of individual patient-level reporting rates depicted in Figure 3 exhibited a bimodal pattern, from which the median cutoff for dichotomization into higher and lower compliance groups was determined. The only variable significantly associated with greater compliance was occupational status, such that women without any kind of employment were associated with a higher rate of compliance (*P*=.03; Table 1). Furthermore, the individual patient-level reporting rate of this jobless subgroup was significantly higher than that of those with some form of employment (55.9%, SD 25.7 and 30.7%, SD 19.2, respectively; *P*=.006; Table 2). Average individual patient-level reporting rates in each subgroup classified according to the clinicopathologic variables are summarized in Table 2. Age, educational attainment, marital status, cohabitation status, and baseline BDI and HRQOL scores were not significantly associated with compliance. This study cohort was clinically homogeneous and the lymph-node status, hormone receptor expression, and chemotherapy regimens did not differ significantly between the two compliance subgroups (Table 1).

**Figure 5:**
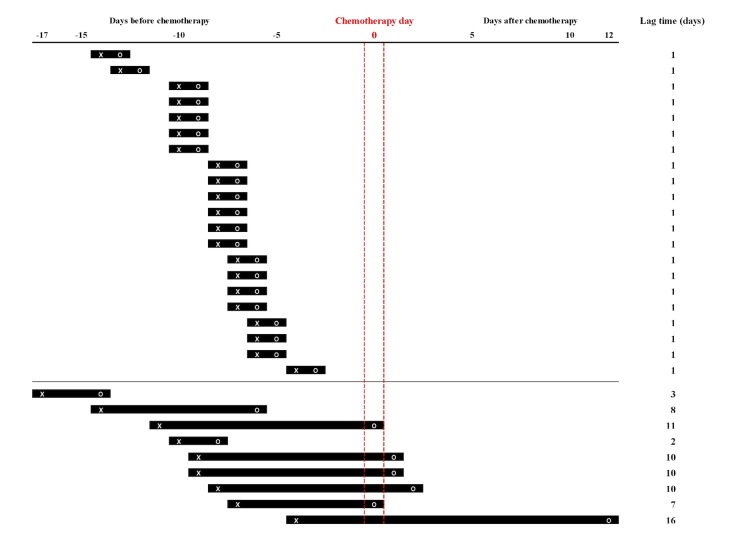
Intervals from enrollment to first self-reporting. X and O indicate day of enrollment and day of start of self-reporting, respectively.

The interval from enrollment to first self-reporting (ie, lag time) was also investigated in this study. As shown in Figure 1, a push notification that asked “Did you check your journals today?” was sent daily to the patients’ smartphones from the day after enrollment and the participants were able to begin their self-reporting via the app from that day at their leisure. There was no additional reminder process via any type of online or offline tool. The intervals from enrollment to first self-report are depicted in Figure 5 (median 1 day; range 1-16 days). The mean individual patient-level reporting rate was higher for the subgroup with a 1-day lag time, defined as starting to self-report on the day immediately after enrollment, than for those with a lag of 2 or more days (51.6%, SD 24.0 and 29.6%, SD 25.3, respectively; *P*=.03).

**Table 2:** Average individual patient-level reporting rates in each subgroup classified according to the clinicopathologic variables (n=30).

Subgroup classified according to each variable		n (%)	Individual patient-level reporting rate (%)		
			Mean (SD)	Range	*P*
**Age**					NS^a^
	Young age (≤49 years)	22 (73)	46.0 (23.8)	7.8-94.4	
	Old age (≥50 years)	8 (27)	42.4 (33.1)	6.7-95.6	
**Level of educational attainment**					NS
	Up to high school	16 (53)	40.3 (24.8)	6.7-95.6	
	College or greater	14 (47)	50.3 (27.3)	7.8-94.4	
**Marital status**					NS
	Married	26 (87)	45.6 (26.4)	6.7-95.6	
	Single	1 (3)	50.0		
	Divorced	3 (10)	37.8 (31.8)	7.8-71.1	
**Cohabiting**					NS
	Yes	28 (93)	43.6 (26.1)	6.7-95.6	
	No	2 (7)	65.0 (21.2)	50.0-80.0	
**Occupation**					.006
	Yes (of any kind)	13 (43)	30.7 (19.2)	6.7-71.1	
	No (eg, stay-at-home mother)	17 (57)	55.9 (25.7)	10.0-95.6	
**Baseline anxiety status with BDI** ^b^					NS
	No anxiety (BDI≤15)	21 (70)	46.2 (27.8)	7.8-95.6	
	Anxiety (BDI≥16)	9 (30)	42.2 (22.7)	6.7-80.0	
**Pain/discomfort status with EQ5D-3L** ^c^					NS
	No problems	19 (63)	41.2 (27.5)	6.7-95.6	
	Problems	11 (37)	51.6 (23.0)	24.4-80.0	
**Anxiety/depression status with EQ5D-3L**					NS
	No problems	14 (47)	49.6 (29.9)	7.8-95.6	
	Problems	16 (53)	41.0 (22.3)	6.7-78.9	
**Disease status**					NS
	Localized	7 (23)	41.6 (31.4)	13.3-95.6	
	Advanced	23 (77)	46.0 (24.9)	6.7-94.4	
**Hormone receptor status**					NS
	Positive	18 (60)	52.8 (24.8)	6.7-95.6	
	Negative	12 (40)	33.2 (24.2)	7.8-94.4	
**Neoadjuvant chemotherapy regimen**					NS
	AC^d^#4	7 (23)	41.6 (31.4)	13.3-95.6	
	AC#4 followed by docetaxel#4	19 (64)	50.7 (24.6)	6.7-94.4	
	FEC^e^#3 followed by docetaxel#3	4 (13)	23.9 (11.1)	7.8-33.3	

^a^Not significant.

^b^BDI: Beck’s Depression Inventory.

^c^EQ5D-3L: EuroQol Five Dimensional Questionnaire.

^d^Combination of doxorubicin+cyclophosphamide chemotherapy.

^e^Combination of 5-fluorouracil+epirubicin+cyclophosphamide chemotherapy.

### Primary Reasons for Missing Self-Reporting of Sleep Disturbance Data

A total of 24 participants responded to the question regarding their primary reason for missing self-reporting. The most common response, which accounted for 38% (9/24) of all responses, was that “The app didn’t work properly.” Other reasons included “I forgot” (29%, 7/24) and “I didn’t think it was useful” (21%, 5/24). Minor reasons were “I was too sick” (8%, 2/24) and “I didn’t feel like it” (4%, 1/24). No missing data were due to either “It was inconvenient” or “I was too busy” (Table 3). As shown in Table 3, average individual patient-level reporting rates in each subgroup categorized according to the reasons were not statistically different.

**Table 3:** Reasons for missing a self-reporting event and average individual patient-level reporting rates (n=24).

Reason	n (%)	Individual patient-level reporting rates (%) mean (SD)^a^
The app didn’t work properly^b^	9 (38)	49.6 (24.6)
I forgot	7 (29)	32.4 (23.0)
I didn’t think it was useful	5 (21)	57.8 (26.6)
I was too sick	2 (8)	42.2 (40.9)
I didn’t feel like it	1 (4)	33.3
It was inconvenient	0 (0)	NA^c^
I was too busy	0 (0)	NA^c^
No response	6	45.0 (31.2)

^a^Means among the subgroups were not significantly different by ANOVA (analysis of variance).

^b^Temporary dysfunctions such as delay or failure of log-on or abnormal shutdown of the app during the self-reporting.

^c^Not available.

## Discussion

### Principal Findings

This study has shown that the collection of patient self-reporting symptoms via a smartphone at daily intervals for a relatively long-term period may be feasible in women with a recent diagnosis of breast cancer and receiving ongoing chemotherapy. The overall rate of compliance was 45.0% and the median value of individual compliance (ie, individual patient-level reporting rates) was 41.1%. About half of the participants did not self-report at around the 5^th^ week after starting to use the app and only 13.3% of the participants could be expected to self-report at the 90^th^ day. We found that in this population, depression, HRQOL status, and demographic characteristics such as age and educational level did not affect compliance, but the results suggest that women who were not currently in employment and those who started to use the app on the day immediately after enrollment exhibited greater compliance with daily self-reporting.

**Figure 6:**
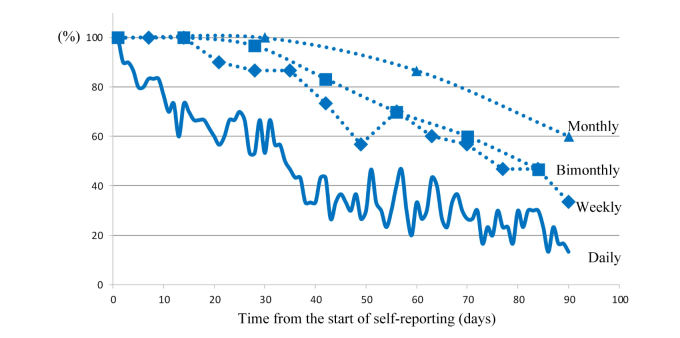
Comparison of longitudinal compliance rates according to different time units.

During the past decade, the greatest upsurge of mHealth research occurred between 2007 and 2008, when the new generation of smartphones, such as the iPhone and similar devices, were introduced [15]. Since most mHealth apps in mHealth research have focused on fields such as chronic conditions [15], followed by prevention/well-being and acute conditions, the evidence around smartphones with apps in cancer patients is extremely limited, particularly regarding the feasibility of nonclinic-based and daily interval data collection, as in this study. Previous studies not involving the use of smartphones found that the completion of online questionnaires on toxicity in patients receiving chemotherapy was associated with a high compliance rate (on average more than 70-80%) for self-reporting at office visits, but only 15.0% actively self-reported from home between visits [7,8]. However, our overall compliance rate was 45.0%, which is higher than found in previous studies, especially considering that the responses originated from the home between clinic visits and the response interval was daily. In addition, our overall compliance of 45.0% was calculated based on daily self-reporting and so can be considered as daily compliance. If patient-level compliance is quantified, defined as the proportion of each time unit during which a given patient self-reported at least once, as in one previous study [9], our weekly, bimonthly, and monthly compliance rates increase to 69.7%, 76.1%, and 82.2%, respectively, all of which are above a reasonable cutoff value by experts (Figure 6) [9]. Previous research indicates that the main barrier to self-reporting via the Internet is not receiving a reminder while at home (ie, participants simply forget) [8,9,14]. We can assume that the regular push notifications available with this kind of app may be responsible for the higher-than-expected compliance rate seen in the present study.

**Figure 7:**
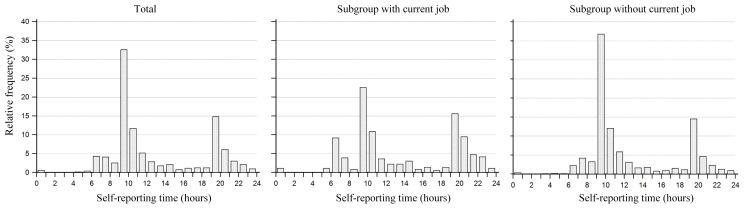
Push notifications and distribution of self-reporting time.

The lower compliance observed among women with a current job may be due to failure to immediately respond to push notifications and simple forgetfulness. Self-reporting during the hour immediately following the push notification (9-10 am and 7-8 pm) accounted for almost half of all responses, which is consistent with the proportion of women in this cohort without a current job. However, in women with current employment, the pattern of self-reporting had two wider peaks (Figure 7). To improve the rate of an immediate response to push notification, various schedules for push notification timing from which patients can choose should be developed into the app in future studies. We cautiously expect that such tailored push notifications will improve compliance in the subgroup of the currently employed, since, as suggested by the data in Table 3, simple forgetfulness rather than being busy was the primary reason for missing self-reporting.

The compliance rate was higher for the subgroup without a lag time in this study (mean 51.6%, SD 24.0 and 29.6%, SD 25.3, respectively; *P*=.03). One possible explanation for this is that the interval between enrollment to the first self-reporting event may be a surrogate variable for self-efficacy, which is defined as the extent or strength of one’s belief in one’s own ability to complete tasks and reach goals [16]. The concept of self-efficacy has been receiving increasing recognition as a predictor of changes and maintenance of health behavior [17], as well as of the psychological attitudes of cancer patients [18]. A general self-efficacy questionnaire was not administered in this study and so we cannot draw any definite conclusions regarding self-efficacy and compliance. Further investigation focusing on self-efficacy rather than conventional patients’ characteristics such as age, educational level, marital status, cohabitation status, and mood—which were not significantly associated with self-reporting compliance in this study—might reveal practical and modifiable predictors for selecting the subgroup with the highest compliance rate in the emerging smartphone-based health care field.

In this feasibility study, we also sought to determine the degree of persistence of compliance and the optimal time interval for self-reporting of daily changing symptoms, such as the sleep-disturbance data found in this study. Although it is not possible to form categorical conclusions regarding these parameters from the findings of the present single study, the results will help toward the development of future strategies for collecting repetitive biodata. Researchers working on smartphone-based health care can expect interactive and responsive communications from 50% of women receiving chemotherapy at the 5^th^ week, from 20-50% between the 6^th^ and 10^th^ week and from just 10-30% thereafter (Figure 4). In addition, given daily collection of self-reporting data via smartphone with an overall compliance of 50%, the entire collection period should be less than 10 weeks, corresponding to a cumulative longitudinal day-level reporting rate of 51.3% (Figure 4). The rationale regarding the dropout cutoff in the Methods section was that we considered it unreasonable to keep encouraging daily self-reporting of symptoms by those who respond on average almost weekly or less. From the cumulative dropout probability curve, we predicted that 10%, 20%, and 21% of participants would drop out at days 28, 56, and 84, respectively (Figure 4).

### Limitations

The limitations of this study include the enrollment only of Korean women with breast cancer at a single urban tertiary cancer center. Other populations may exhibit different self-reporting characteristics in terms of compliance and should be independently evaluated. In addition, these findings should be generalized only after taking into account that this study was conducted under special circumstances in South Korea in which there is easy and ubiquitous access to wireless networks and the penetration of smartphones is the second highest in the world, after the United Arab Emirates (Multimedia Appendix 1). The study design meant that only patients possessing a smartphone with app avidity were included. Of the total population of patients who were approached regarding participation in this study, 13% (9/67) were excluded by this criterion. Therefore, the results of the present study should be interpreted with caution: the compliance rates in this population may have been overestimated. Nonetheless, this feasibility study provides an initial understanding of the opportunities for successful smartphone-based collection of real-time, self-reporting data, and valuable insights into the development of more practicable interventions with smartphones in the real cancer care setting.

### Conclusions

In conclusion, the findings of this study suggest that 90-day, longitudinal collection of daily self-reporting sleep-disturbance data via a smartphone app is feasible. Further research is needed to determine how to sustain the compliance with a self-reporting program over a longer period of time and to select subpopulations with higher compliance rates for mobile health care.

## Multimedia Appendix 1

Top 15 countries with highest smartphone penetration in 2013 (from our mobile planet by Google).

## Abbreviations

app: application
BDI: Beck’s Depression Inventory
eHealth: electronic health
ePROs: electronic PROs
EQ-5D-3L: EuroQol Five Dimensional Questionnaire
HRQOL: health-related quality of life
ICT: information and communication technology
mHealth: mobile health
OS: operating system
PRO: patient-reported outcome
