# Supplementary figures and images for: Daily Collection of Self-Reporting Sleep Disturbance Data via a Smartphone App in Breast Cancer Patients Receiving Chemotherapy: A Feasibility Study
Source: J Med Internet Res. 2014 May 23;16(5):e135. doi: 10.2196/jmir.3421 (PMC4051741; doi:10.2196/jmir.3421)

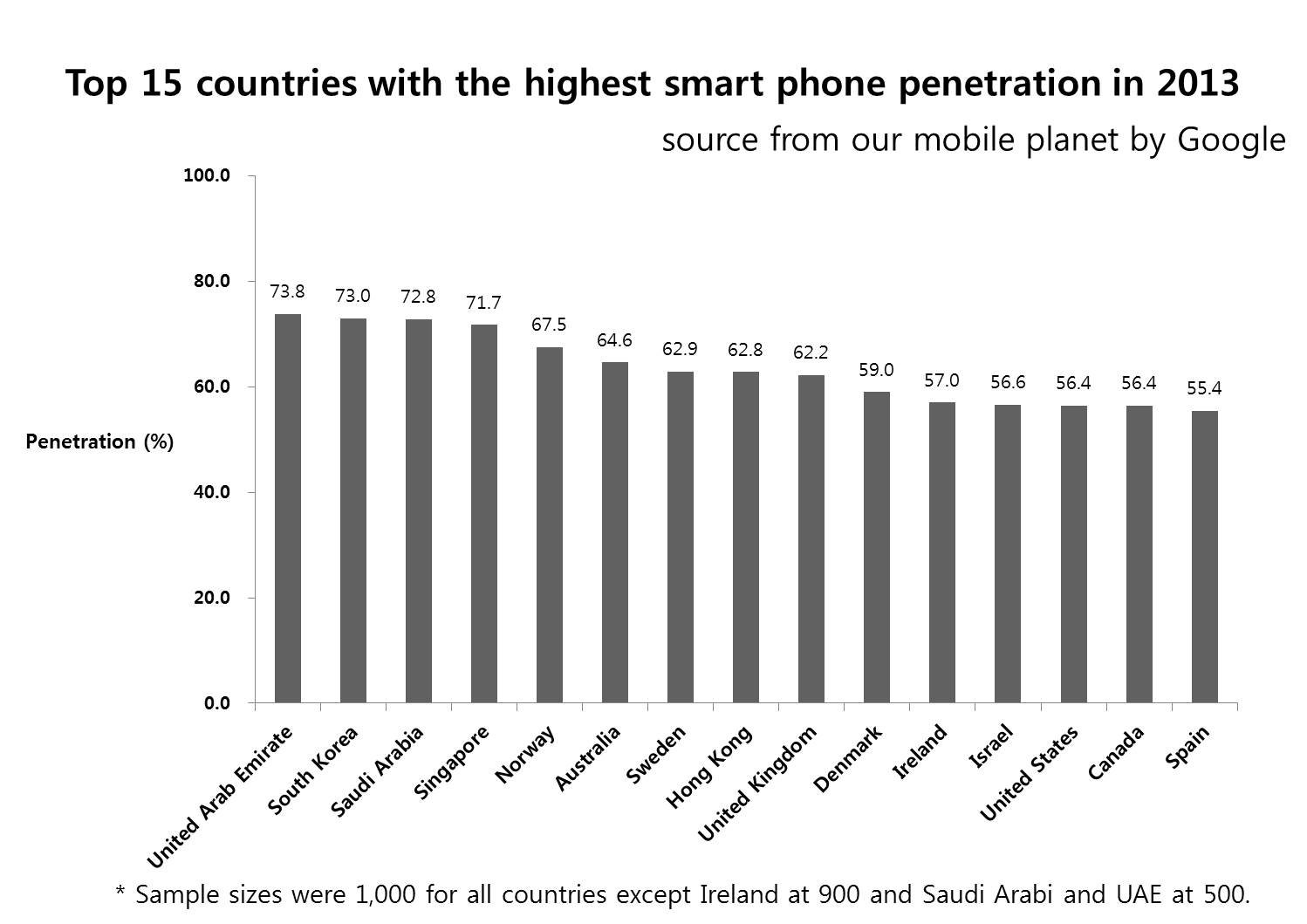

Supplement: Supplementary file 1 [file jmir_v16i5e135_app1.jpg]
